# Supplementary figures and images for: ETV2 Upregulation Marks the Specification of Early Cardiomyocytes and Endothelial Cells During Co-differentiation
Source: Stem Cells. 2022 Dec 13;41(2):140–52. doi: 10.1093/stmcls/sxac086 (PMC9982073; doi:10.1093/stmcls/sxac086)

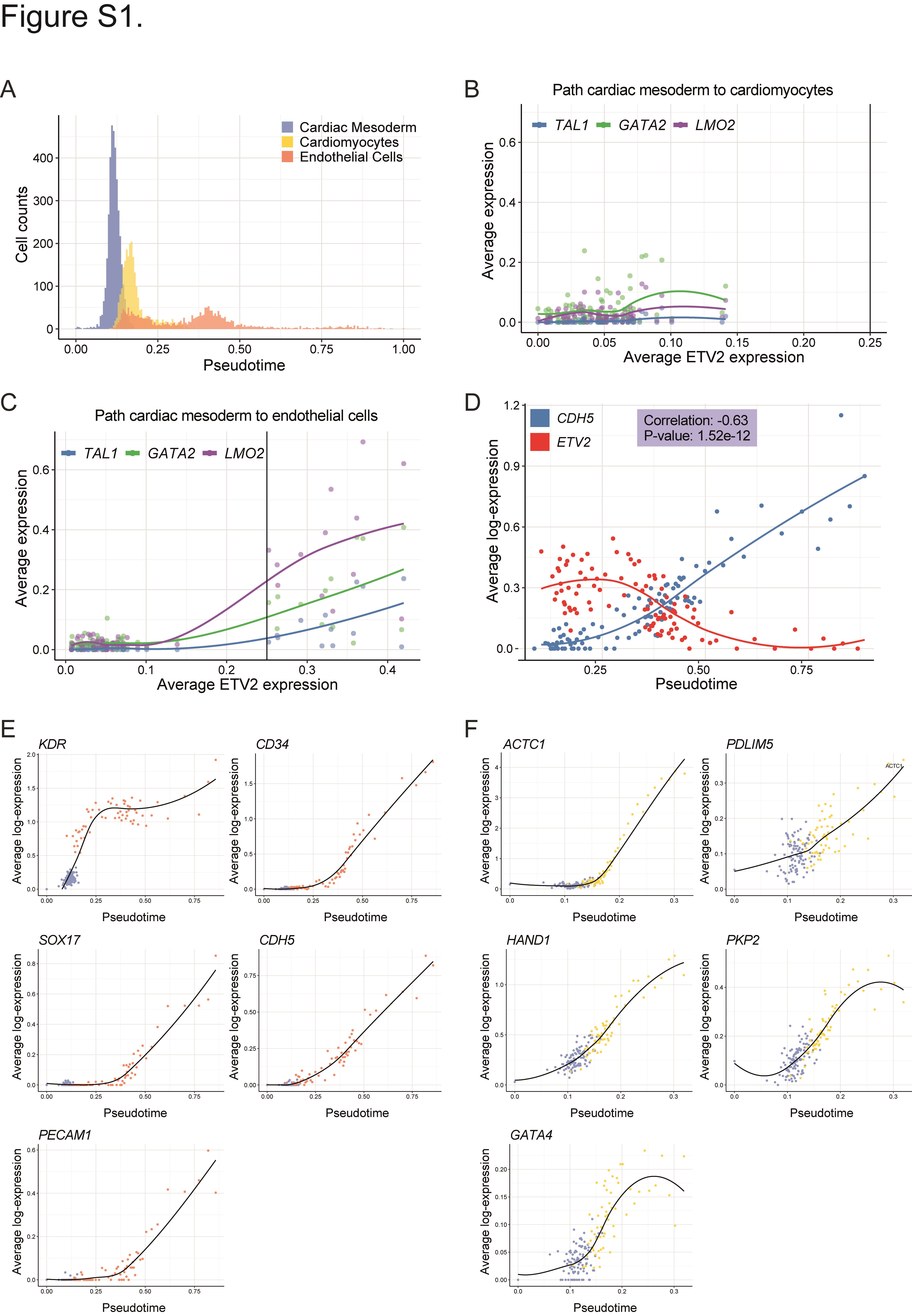

Supplement: sxac086_suppl_Supplementary_Figure_S1 [file sxac086_suppl_supplementary_figure_s1.jpeg]

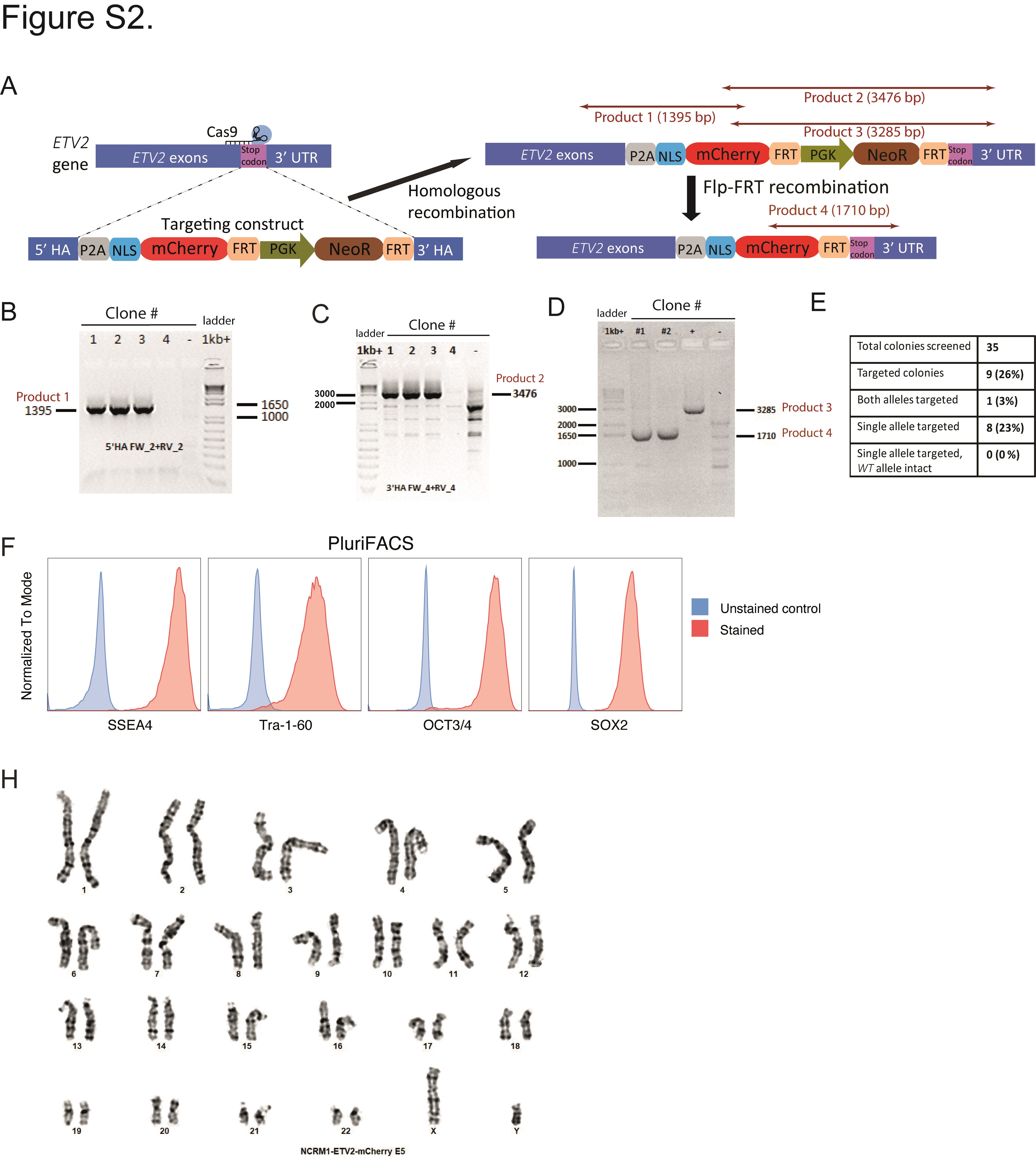

Supplement: sxac086_suppl_Supplementary_Figure_S2 [file sxac086_suppl_supplementary_figure_s2.jpeg]

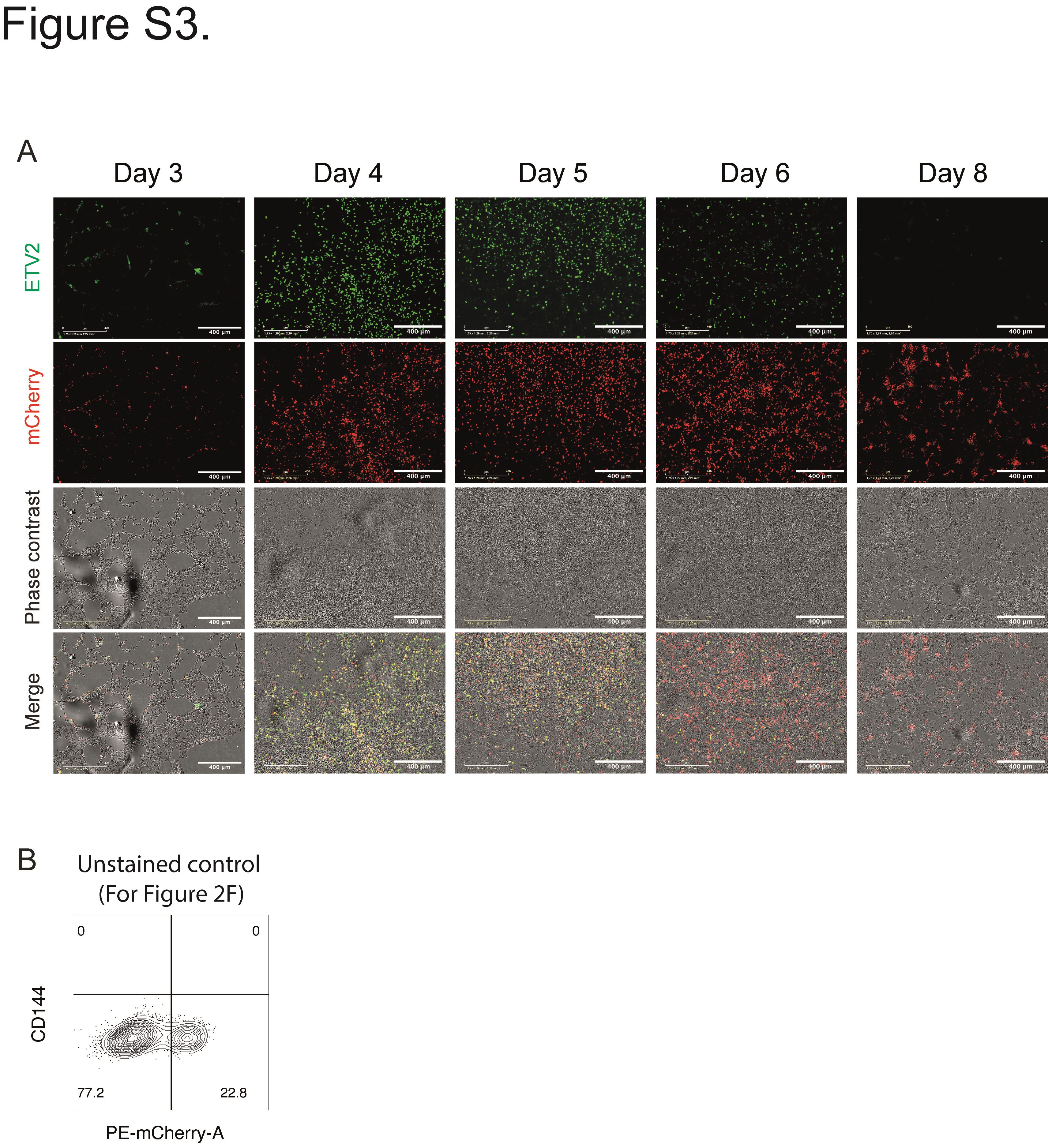

Supplement: sxac086_suppl_Supplementary_Figure_S3 [file sxac086_suppl_supplementary_figure_s3.jpeg]

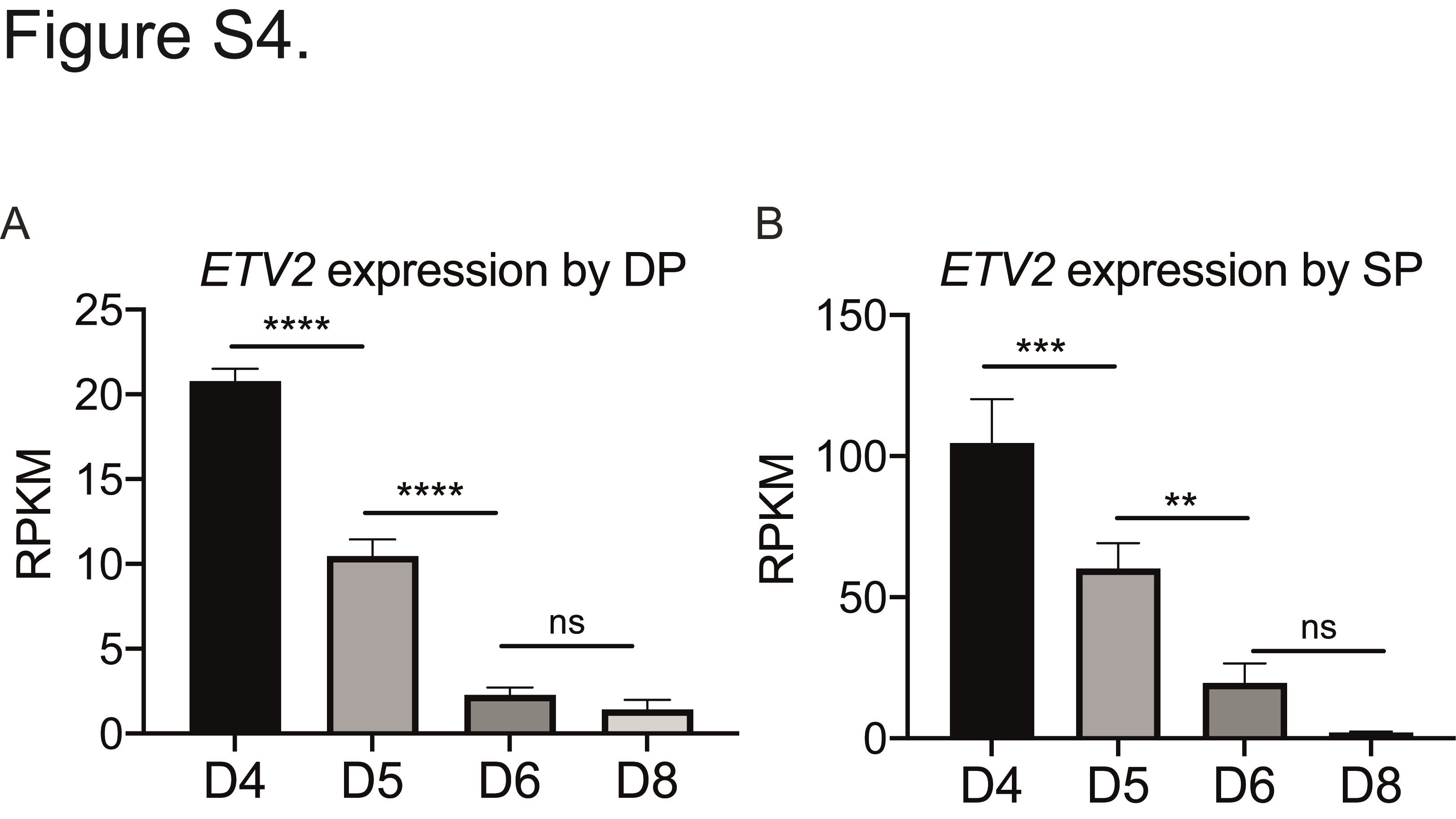

Supplement: sxac086_suppl_Supplementary_Figure_S4 [file sxac086_suppl_supplementary_figure_s4.jpeg]

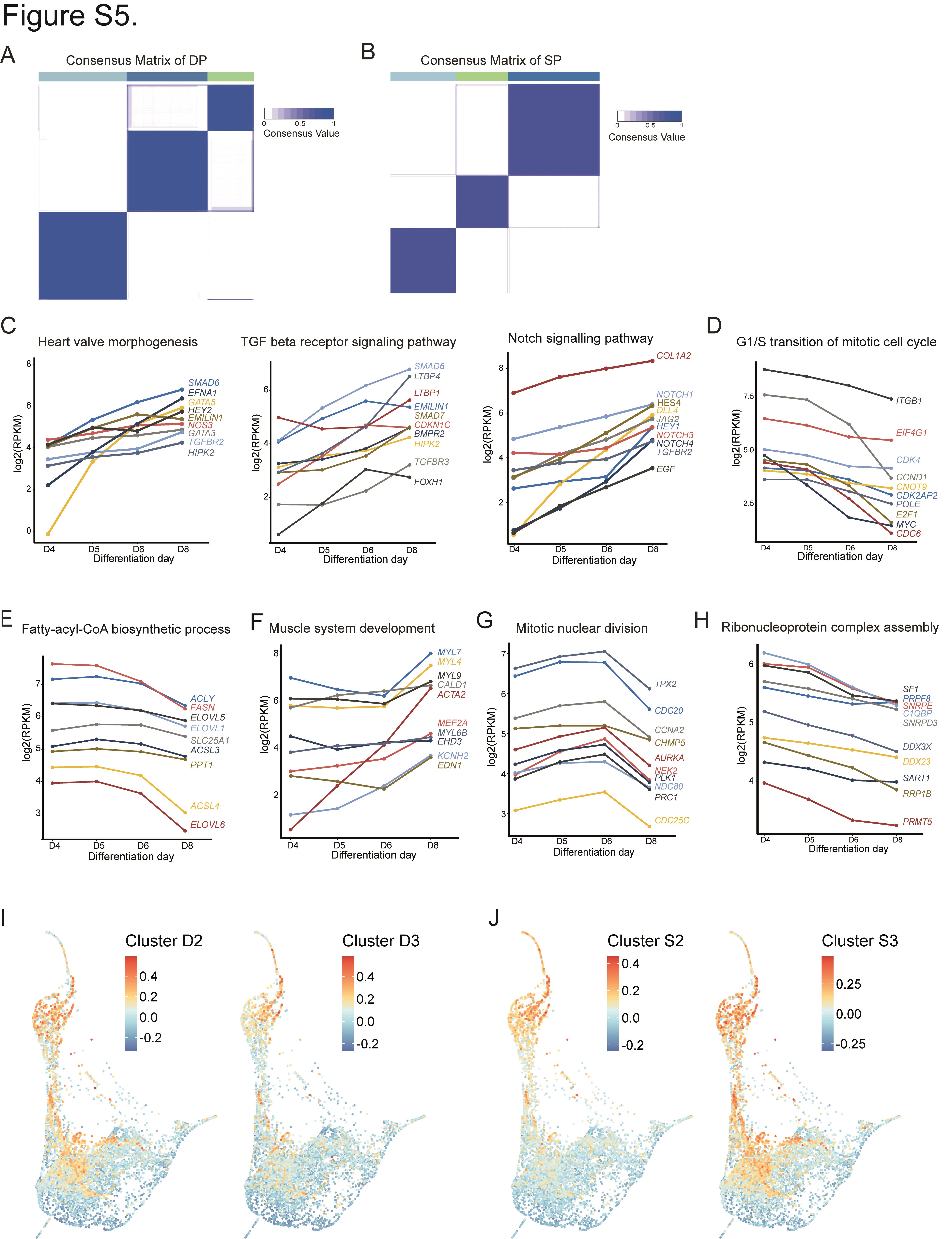

Supplement: sxac086_suppl_Supplementary_Figure_S5 [file sxac086_suppl_supplementary_figure_s5.jpeg]

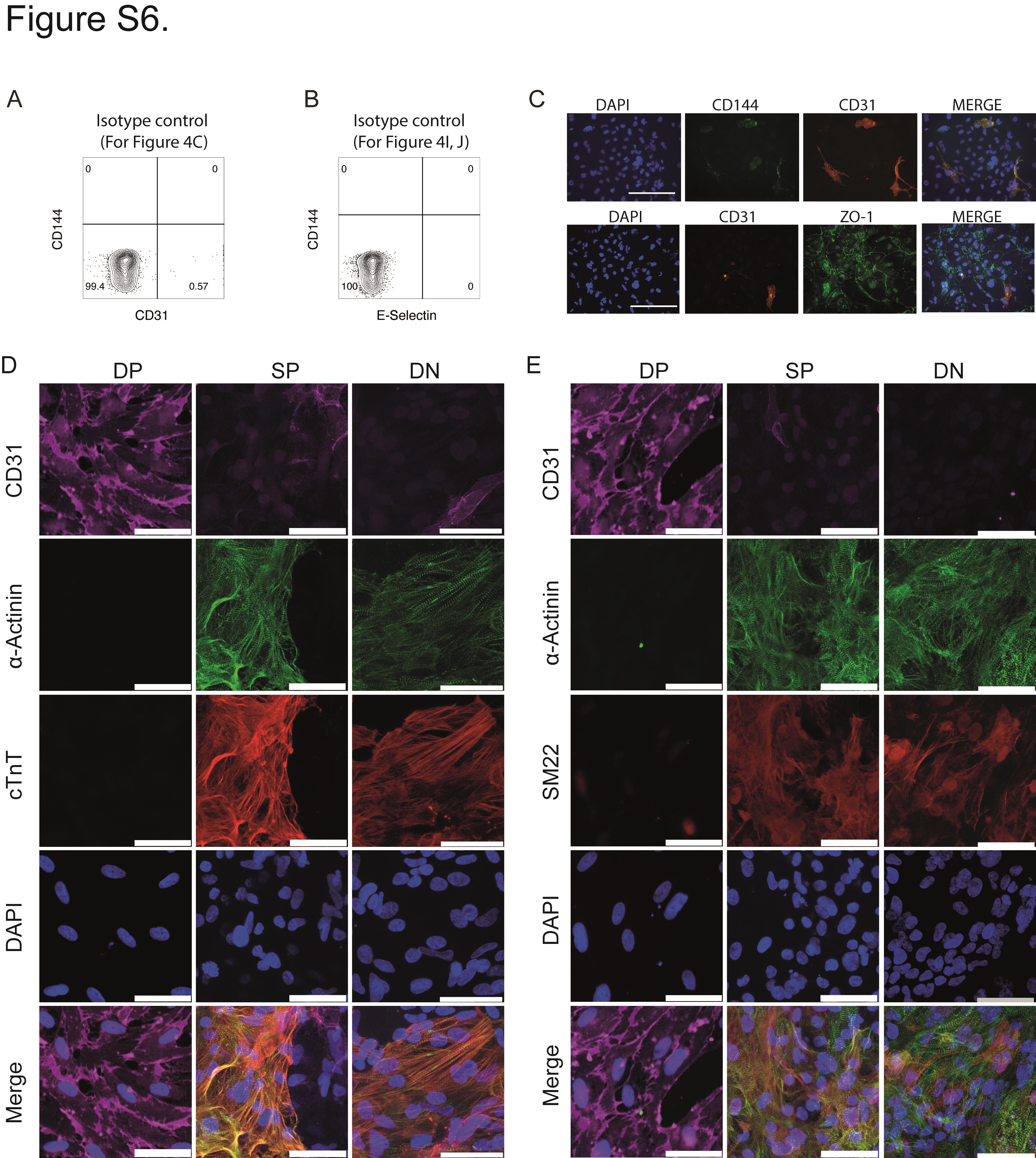

Supplement: sxac086_suppl_Supplementary_Figure_S6 [file sxac086_suppl_supplementary_figure_s6.jpeg]
